# I Am a Nursing Student but Hate Nursing: The East Asian Perspectives between Social Expectation and Social Context

**DOI:** 10.3390/ijerph17072608

**Published:** 2020-04-10

**Authors:** Luis Miguel Dos Santos

**Affiliations:** Woosong Language Institute, Woosong University, Daejeon 34514, Korea; luisdossantos@woosong.org

**Keywords:** career development, counselling, cultural perspective, decision-making process, East Asian perspective, filial piety, nursing education, nursing shortage, nursing student, turnover

## Abstract

From the East Asian social and cultural perspectives and contexts, this study aimed to understand the relationships and behaviors between nursing students’ sense of filial piety and their decision-making behind selecting nursing education as their major. Forty-two traditional-age nursing students (i.e., six men and 36 women) at their final year of a bachelor’s degree program in nursing were invited. The findings indicated that many nursing students disliked their university major and the potential career pathway as a nursing professional, as none of them selected the major based on their choice and interest. The environmental context and family’s recommendations were the major impacts to influence the decision-making process of the participants. The result also indicated that filial piety, parents’ recommendations, and elderly people’s suggestions were the key factors to influence the selections and decisions of university major and career development pathways. The study provided a blueprint for related staff and professionals to create and design career counselling and services for East Asian youths to enable life investment and development.

## 1. Introduction

Nursing education and training are popular university majors for secondary school graduates, university students, and even adult returning students wishing to pursue life-long personal investment and development [1]. Every year, a large number of secondary school graduates and returning students decide to study nursing as their university major and to develop their careers. Although many individuals start their university education directly after completing secondary school, there are also a large number of adult returning students deciding to start their college and university study during mid-adulthood [2]. The selection of one’s university major and career development path are decisions that impact the rest of one’s life [3]. However, individuals’ social, family, personal, educational, and economic patterns influence their decision-making processes, particularly for East Asian people, who have a strong sense of collectivism [4]. The expression of decision-making for career development is more likely employed when describing that process, which can happen at different stages of life [5].

Although nursing education is a very famous and popular university major, an inappropriate matching and mismatching of career development continues to happen in this profession. One study [6] collected information from 648 questionnaires at three hospitals in Taiwan about nurses’ job satisfaction and intention to quit their position. The results indicated that a large number of nurses planned to leave their positions due to significant levels of stress and burnout at their workplace. Although they understand their job natures and responsibilities before entering the profession, many nurses decide to switch their career profession due to ideas of balancing work and family [7].

One study [8] employed the Ward Organizational Features Scales (WOFS) to measure the relationship between job satisfaction and work responsibilities of 834 acute ward nurses in the United Kingdom. The relationship between nurses and other medical staff was one of the most critical elements for their career decision. Additionally, many nurses indicated that the workload of their positions always negatively impacted their job satisfaction due to unbalanced responsibilities. It is worth noting that the negative measurement affected the career decisions of these nurses, who worked in a stressful environment [9].

A recent study [10] about the relationship between disappointment and nursing student retention collected data from 17 nursing students during the third year of their nursing program at university level. The report indicated that many nursing students decided to join the nursing profession because of the desire to provide a caring service, a personal background with the healthcare profession, modelling from peers, and potential career advancement. One study [11] indicated that a number of nursing students explained that negative experiences regarding their training, teaching staff, placement experience, and teammates always affected their career decisions. For example, some participants indicated that the excessive responsibilities and expectations from their supervisors could influence their career decisions due to negative placement experiences. Based on the results, it is not hard to predict that many nursing students or fresh graduate nurses may leave the nursing profession within the first few years of their career [11].

Another study indicated that nursing programs always have a high dropout rate due to the unique nature of the profession [12]. Unlike doctors who can provide individualized medical treatments to patients, nurses tend to assist other medical professionals. In other words, nurses cannot offer individual services without the supervision of other medical professionals. Therefore, once nursing students understand the nature of the profession, many decide to drop out [13].

Expectation is another element of dropout [14]. Providing a caring service is meaningful for many individuals who want to join social care and health services. However, the responsibilities of nurses are unique. Unlike social workers and counsellors who provide counselling and mental health services to individuals with problems, nurses need to provide both psychological and physical labor services from cleaning to operation room assistance. Therefore, the responsibilities are more comprehensive than for many professions in the field of social care and healthcare. As a result, after students understand the relationship between the duties and their expectations, they may drop out or leave the profession [15].

However, many individuals decide to enter the nursing profession due to stable job advancement and salary, particularly in developing countries [16]. Nursing, teaching, and social work are three demanding professions in society internationally. Many enter these fields due to financial, family, and personal considerations. Although these professions always welcome learners and second-career professionals, inappropriate matching is not uncommon. However, for various reasons, some mismatched individuals never leave their positions. In such cases, low-level performance, motivation, and morale prevail in the workplace [17].

One study [18] indicted that East Asian students are more likely to select a university major based on the interests and expectations of their parents, while most European students tend to select their major based on their own interests. Jin [19] indicated that East Asians’ major selection tends to be influenced by filial piety toward parents. The researcher employed the Career-Related Filial Piety Scale (C-FPS) to investigate the relationship between parents’ decisions and students’ choices. The results indicated that students usually tend to study at universities their parents like and enter professional fields according to their parents’ preferences. More recently, a study [20] conducted in Hong Kong reflected this as well. The study collected information from 522 undergraduate students about their reciprocal and authoritarian behaviors and senses about the relationship between students’ filial piety and career decisions. The results indicated that although many students select their career pathways based on their interests, most respect their parents’ wishes according to the traditional East Asian perspective [21]. Perhaps the social and cultural environment in Hong Kong influences young adults’ career decisions due to the westernized nature of that society; most Chinese students and participants tend to listen to their parents in order to avoid arguments and misunderstanding [22].

### Purpose of the Study

The researcher has conducted research in the fields of social care, human resource management, school administration, and organizational psychology. The personnel shortage in the areas of social caring and nursing has been an issue for decades. Although many human resource professionals, policymakers, school administrators, leaders of social care departments, and researchers always establish plans to encourage potential medical professionals to work in the field. However, the results of these plans were not always effective. Due to the social and cultural expectations, the researcher wants to understand how the East Asian perspective influenced these issues. Currently, studies with a focus on nursing education and nurses’ career development tend to explore participants’ elements from a westernized perspective [7,11,13,16], in which individualism is the primary social norm and practice [4]. Therefore, this study aimed to understand the relationship between East Asian nursing students’ sense of filial piety and their decision-making behind selecting nursing education as their major [23,24,25,26,27,28,29,30,31,32,33,34].

There are two purposes of this study. First, every year, a significant number of students decide to enroll in nursing education programs for initial training. Based on the literature review, various reasons for this have been found among these students [7]. However, many of them drop out of the programs or leave the nursing profession within the first few years of their career development [14]. Therefore, this study sought to study a group of traditional-age nursing students enrolled in a nursing education program but who will not enter the nursing profession after graduation. Second, the study sought to understand the factors that contribute to the relationship between East Asian university students’ filial piety [20,35,36,37] and their decision-making behind selecting their university major and career development pathway in nursing [38]. The elements studied included outcome expectations, interests, and goals [38].

As a result, little empirical evidence exists for East Asia regarding the relationships between expectations, social-economic backgrounds, social environmental factors, and personal interests of young individuals and their selection of university subjects and career paths [8,9,10,11]. It is worthwhile to note that, unlike in Western cultures, Asian young people tend to highly respect their parents’ suggestions, as well as the recommendations of their elders, such as parents, grandparents, teachers, and elder siblings, due to traditions of filial piety [8]. In short, this study serves as one of the first attempts to understand the relationship between filial piety and the decision-making of East Asian university students regarding their major selection and career development [23,24,25,26,27,28,29,30,31,32,33,34]. Unlike with Westerners, the values of filial piety, collectivism, and sense of family union are essential elements for East Asian people [39]. Therefore, this study will help provide opportunities for readers, university administrators, international school staff, educators, and international student service professionals to establish effective counselling services to assist this particular group of students.

## 2. Materials and Methods

The employment of qualitative research methods [40] would be appropriate. Unlike quantitative research, qualitative research method allows the researcher to collect rich and in-depth data information from the participants. The researcher could access the information and lived stories which could not be answered by statistics and numbers [41]. Although the researcher had aimed to collect information from one single university counselling centre for a case study, the researcher believed the data information from one single source might not be able to express the holistic pictures of the current problem. Therefore, the researcher decided to employ the general qualitative approach [40,42,43,44], which may better apply to this study. Thomas [44] indicated that the general qualitative approach is an inductive approach that may meet most of the requirements for qualitative researchers. Moreover, without additional requirements, qualitative researchers could freely collect data information from the targeted groups. As the current study might not fit other qualitative methods, such as case study, the general qualitative approach would be the selection [41,44].

### 2.1. Participants

Fort-two traditional-age nursing students (i.e., six men and 36 women) at their final year of a bachelor’s degree program in nursing were invited. All agreed to participate in this study. All the participants were local Taiwanese students. Five out of the 42 were indigenous Taiwanese. All were born and raised in Taiwanese families with the traditional East Asian conceptions. The age range was 18-22 years. The researchers invited these participants with the snowball sampling strategy [40,42,43]. In other words, the researcher invited participants based on the networking from other participants. Participants were enrolled at five different universities in Taiwan. The participants needed to meet the following criteria, which were:

1. he/she is currently enrolled at a nursing education program in Taiwan;
2. he/she is currently enrolled in the final year of the program;
3. he/she was born and raised in Taiwan;
4. he/she needed to meet the age requirement.

### 2.2. Data Collection

Two types of tools were employed, including individual and focus group activities [40,43,45]. The individual interviews were conducted to explore their understanding, life experience, and family issues under a private sharing environment [46,47,48]. The focus group activities were conducted to explore some similar background, social and family expectations, social bias, and interests. Both research tools were particularly useful in regard to listening to the voices by engaging them individually and collectively.

All participants have voluntarily participated in this study. The general inductive approach [44] was employed for qualitative data collection and analysis. The researcher was the collector of the data information. In order to seek meaningful data information, first, the semi-structured interview sessions were created. According to Seidman [49], individuals are less likely to share lived stories and personal background to others without any prior relationships. Therefore, in order to overcome this issue, the researcher decided to conduct two sessions of semi-structured, one-on-one, and private interviews in a private room at a community centre for each participant. Each interview lasted up to 40 min. One of the directions of this study was to explore the direct effect of filial piety on a career decision. Therefore, the protocol interview questions for both individual interview sessions tended to focus on the relationship between an East Asian perspective and the social context and issues. Appendix A shows the protocol interview questions for both interview sessions.

Second, after each participant completed the individual interview sections, all participants were invited into six focus group activities, each with seven participants. As the schedule of each participant was different, the focus group activity hosted seven members regardless of their university enrolments. The focus group activities were hosted at the same community centre. Appendix B shows the protocol interview questions for the focus group activities.

As it might be difficult for participants to share their understanding and experience in English language and a language other than their mother tongue, all the sections were conducted in Chinese Mandarin and translated into English. All the interview and focus group activities were audio-recorded and transcribed into written documents for data analysis. Member checking was done after the data analysis procedure. In order to protect the personal rights of each participant, all participants were given a pseudonym.

### 2.3. Data Analysis

After the data collection procedure, 543 pages of written transcripts were created based on the semi-structured interviews and focus group activities. Qualitative researchers [40,43,45] advocated that large-size data information should be narrowed down to meaningful themes and patterns. Therefore, the researcher followed the general inductive approach (GIA) [44] for data analysis. First, the researcher used the open-coding procedure for the initial themes. After the initial themes were formed, the axial-coding procedure was employed for the second-level themes. As a result, two themes and seven subthemes were merged.

### 2.4. Human Subject Protection

All the signed and unsigned agreements, personal contact, audio recording, written transcripts, computer, and related materials were locked in a password-protected cabinet. Only the researcher had the means to open it. After the study was completed, the researcher immediately destroyed and deleted all related materials for personal privacy.

Due to the agreement, the university information and place of origin was masked due to privacy. Most of the students were concerned that their university information would be disclosed. Due to the small population and closed professional networks, the researcher needed to protect the information of the participants. However, the participants allowed the researcher to show their gender for this study. Therefore, the following part will outline their gender with their discussion. It is worth noting that a small number of participants agreed to disclose their place of origin. The researcher reported this information with the discussion of the findings.

All subjects gave their informed consent for inclusion before they participated in the study. The study was conducted in accordance with the Declaration of Helsinki, and the protocol was approved by the Ethics Committee of The Youth Caring Association (2018/2019/SummerFall).

## 3. Findings and Discussion

During each interview and focus group activity section, the participants answered the same general semi-structured questions that asked for their opinions and feedback. Although all participants had a similar family and social background in the same country, having come from one of the Taiwanese areas, their personal sharing, lived stories, and life experiences were not the same. Unlike in other studies, which have focused on westernized cultural perspectives, all participants reported that their major selection (i.e., nursing) was influenced by their family members, parents, and social expectations. In order to help answer the research questions, the findings were categorized into two superordinate themes and seven subthemes. It is surprising to note that all 42 participants were studying a university major that was not their own selection or interest. In other words, upon graduation, they would receive a degree, initial license, and potentially develop their long-term career pathways in a direction in which they have no strong interests. More importantly, many expressed that they will leave the nursing profession after graduation. Table 1 outlines the themes and subthemes of this study.

### 3.1. Influences from the Participants’ Contextual Environment

Before the session began, the researcher needed to indicate that the statements about I hate nursing. I dislike the medical career had been recorded 356 times. It was very surprising that the mismatching of major and human resources (i.e., students) was significant in the current Taiwanese university environment, particularly in the faculty of nursing. Although the current study could not represent the overall situation in Taiwan and the East Asian region, the significant results from this study may explore and contribute to an understanding of how these extreme situations [14] happen in the East Asian region [31].

By listening to the stories and life experiences of the participants, the researcher identified several elements that impacted their major decisions. The findings of this study discovered that all did not follow their personal goals and interests when selecting their major (i.e., nursing). The results reflected that the participants’ behaviors also tended not to follow their own interests in choosing a career, which goes against a large number of previous findings [7,15,16]. It is worth noting that in most cases in East Asia, university students are not allowed to switch their major once they have been accepted. In other words, students are required to complete the major and degree that they submitted as their choice with their application. Therefore, the contextual and environmental factors that prevailed during their secondary school period highly influenced their life-long developments and career decisions [50]. A group of participants expressed that nursing was a choice of their parents during the application period, saying, “I can apply up to six universities and majors…But once I admitted with a school and major…I cannot switch…but the major application was written by my mother without my agreement…” (P#5, Male, Interview).

The results of this study supported the idea that youth have their own ways of thinking about career developments [7,51,52]. However, because the university’s constraints on their university decisions and their overall contextual factors limited their opportunities, most were unable to exercise the career pathways that they might have decided on their own [14]. A discussion of the influences on the career decisions of the participants follows.

#### 3.1.1. The East Asian Perspective on Occupational and Role Expectations

All 42 participants expressed that the East Asian perspectives about occupational and role expectations influenced their decisions about the major they were studying, their university enrollment, and their career decisions [53]. In the traditional East Asian perspective, medical practitioners are considered to be upper-class citizens with a high level of social status [54]. Although more than half of the participants expressed their desire to become an artist, reporter, chef, journalist, or photographer, none of them selected the majors associated with those occupations. One participant expressed that almost all artists and performers in the fine arts could not become famous until their death, saying, “Although I want to be an artist, I may become a nurse and do some part-time art activities…But I am not sure will I become a nurse become I hate this occupation now…” (P#2, Male, Focus Group). Another participant (P#4, Female, Interview) believed her hobbies in dancing could not be a long-term occupation, sharing,

I chose nursing was because I think medical professionals are very smart in our society…I really enjoy the perspective of medical professionals…Although I want to become an artist with my wish and interests…both athlet[e]s and dancers can only work in their occupation until the early 30s. After that, the lucky one[s] can work as [a] coach. The others are living under the poverty line….but now, I don’t know…should I become a nurse? I hate this career….

(P#4, Female, Interview)

It is worth noting that not only these two participants, but all 42 participants also expressed the ideas *I hate nursing and I dislike medical professions* in this category. Many expressed interests in other fields but had no chances and opportunities. Although the participants tried to escape from the major (i.e., nursing) they were not interested, they still needed to complete the degree and training due to the East Asian perspective and expectation from the society.

The lack of long-term career development in Taiwan always prohibited youths from joining their dream professions [55], particularly in the humanities and fine art occupations. A participant (P#24, Male, Focus Group) shared an idea:

I like[d] botany and plant biology from [a] young age. I wish[ed] I c[ould] become a botanist from 8 years ago…[but] the long-term development of [a] botanist is unclear. No one can guarantee jobs after graduation, as there are more than 3,000 graduates in this field yearly. Also, Taiwanese people don’t really consider Botanist as a professional. So if I can, I [will] select nursing instead. At least the general public will consider doctors as upper professionals….

(P#24, Male, Focus Group)

It was not surprising to hear that in East Asia, university subject selections were not based on the students’ own interests and desires for career development [23,24,25,26,27,28,29,30,31,32,33,34]. For example, the above participants wished to join botanical biology during her secondary school period. But due to the social context and social expectation [39], she needed to select the alternative (i.e., nursing) as her sixth choice on her application. One participant (P#36, Female, Interview) shared about enrolling in a nursing school instead of an electronic engineering program based on the gender-oriented bias and social expectation of gender-oriented occupations, saying,

…my parents and the society…in East Asian countries believe engineers, doctors, pharmacists, and emergency medical technicians must be males. I love science and engineering…I want to become a medical engineer in the future…but I mother told me that in many Taiwanese universities, there are only a few female students in any of the engineering programmes…it is ugly to be the only girl….

(P#36, Female, Interview)

Most described their selection of their university major as having been significantly influenced by the East Asian perspective, social context, and social expectations [23,24,25,26,27,28,29,30,31,32,33,34]. More importantly, the sense of collectivism [4] was highly represented in these groups of individuals. For example, several of the female participants refused to study one of the STEM subjects because of the social norms and social expectations of men in these areas. Female individuals, however, were expected to study and join the workforce in lesser industries, such as nursing, administration, elementary school education, and social work [56]. Due to the strong East Asian perspective, social context, and social expectation on selecting an academic major and career, it is worth noting that some young adults might give up on following their own interests merely on the basis of the enrollment of students of the opposite gender in a program for which they had prepared for more than four years [19].

#### 3.1.2. Academic Results

Standardized exams and university admission placement tests are widely used in many countries [57]. In Taiwan, a widely used standardized exam is the General Scholastics Ability Test (GSAT). Although there are alternative pathways for enrollment, the GSAT is the most common admission exam for local Taiwanese students. Nearly 30 of the student respondents expressed that their low GSAT scores prohibited their enrollment in the university of their choice and also their selection of university subjects. Unlike in the American educational system, with the community college options, students are allowed to select an undecided major pathway. But Taiwanese students have to select their university enrollment and major during their final semester of 12th grade in secondary school. One participant (P#38, Female, Focus Group) expressed,

I wish I c[ould] follow my will to become a medical doctor. But my low score…I can only apply for the nursing program…However, I cannot enrol at a community college or vocational college because there are no ways to build-up to the medical doctor’s programme in the future…If I want to study but not re-take the exam for one more year, the only selection would be nursing…But again, I hate nursing…because it is not the same….

(P#38, Female, Focus Group)

It is less likely for students to switch their university major once enrolled in many East Asian contexts due to unbalanced admission requirements and university policies. Unlike universities in the United States, students started their general education requirement during the first two-year of university education. Except for in special circumstances, students can switch and re-design their university major after the first or the second year of university education. The following participant (P#10, Male, Interview) shared his negative stories about the switching issue in Taiwan, saying,

Once I studied nursing during my first year at university, I discovered that I don’t like this subject. However, in order to change my university subject, I have to be [among] the top-rated students for the purpose of switching. Otherwise, I have to re-apply for the entire GSAT steps again…I hate nursing, I hate it so much…But I cannot switch it due to the administrative requirement…Also, the social expectation of my nursing career…What can I do….

(P#10, Male, Interview)

In addition to their university enrollment and their selection of a major, the participants’ academic results also limited their selection of university location. Some reported that they were forced by their parents and teachers to go to an urban university due to their high score of GSAT. One participant (P#11, Female, Interview) reported,

I was partially willing to study nursing actually if I could stay in my hometown…In the southern part of Taiwan, there is a very good university and the nursing department. The enrolment of this university is my goal. But I received a high score in my GSAT. Therefore, my mother and school principal forced me to come to the capital city with a university that I dislike. I don’t like the university, the social expectation of the university, and how people believed this university should create good nurses…So I hate nursing because of the social expectation….

(P#11, Female, Interview)

It is worth noting that this participant (P#11, Female, Interview) was willing to study nursing if she could stay in her hometown. However, the East Asian perspective, social context, and social expectation [23,24,25,26,27,28,29,30,31,32,33,34] about her testing score, university, major, and potential career development destroyed her career perspective and beliefs. Although she wanted to become a nurse, she will now suffer due to the stresses and pressures from all different directions of her life.

Almost all described their GSAT scores, regardless of the level, as being a factor in their university enrollment, the decision about their academic major, and career development. Because the GSAT score was the most significant element, the decision about university subjects, the location of the university, and even the ability to switch university subjects after enrollment were not controlled by the participants. However, more importantly, a large number of participants expressed that based on their academic results, their parents, teachers, and even school leaders [35] expected them to enroll at top-tier universities and in a specific academic major. Almost all participants were forced to study in an academic major based on the recommendations from their parents and school staff. For example, a participant (P#32, Female, Focus Group) said, “I cannot cho[ose] which school and which location [where] can I study, I have to listen to my parents and teachers”.

#### 3.1.3. Financial Influence

Everyone experienced the stress of financial factors. Due to the East Asian traditions and cultural perspectives [34], children are responsible for taking care of their parents and even grandparents after their university graduation. Although the Taiwanese government does not regulate any policies about birth control, many contemporary Taiwanese families have fewer than two children.

Nearly two-thirds of the participants expressed that they would be the one who would take care of their parents and even grandparents after graduation, so they needed to select a career pathway that would allow them to make a significant amount of money. In fact, such East Asian perspectives on family engagements and responsibilities highly influenced the decision-making processes of these participants [31]. One participant (P#33, Male, Focus Group) told the researcher that being a nurse was almost the only way to take care of his parents and four grandparents after his graduation. In the same focus-group activity section, another participant (P#35, Female, Focus Group) echoed that situation with her own, saying,

In many families, taking care of parents [is] expected. My parents expected me to take care of their late [years]…although I wanted to study music…I must study nursing, [in] which [I] can make money…But this must not be my wills…I really hate nursing…I don’t want to become a nurse after university…I want my life back as an adult after university…I did not tell my mother about this yet…But I am sure I will not become a nurse…I dislike nursing not matter what….

(P#35, Female, Interview)

Another Participant (P#25, Male, Focus Group) grew up in a rural community in central Taiwan. He also identified this family expectation and financial difficulties as a villager in the countryside. As mentioned above, many East Asian elderly were expected to be cared for by their children. Therefore, as a male child, the participant felt a need to send financial resources to his parents after university graduation. Consequently, this participant considered nursing as one of the occupations that could provide financial security, saying,

…my parents sent me to university due to financial stress. I wish I c[ould] work in my family-run farm. But my parents want me to work in an upper social occupation…I need to listen…Moreover, my mother is a disabled person…I need to take care of her after I finished my university…But I want to continue my study in physical therapy in the future…I like the medical profession, but I surely dislike nursing as my life-long career development…I will explore a master’s degree or another qualification soon….

(P#25, Male, Focus Group)

It is worth noting that this participant has a very strong sense in the field of the medical profession. Although he took nursing as his alternative during his university application period, he will continue his career development in physical therapy due to these personal goals and financial considerations.

In addition to their concerns about post-graduation financial stress, participants also experienced pre-graduation financial stress due to their lower-income status. One participant (P#9, Female, Focus Group) used to plan to study in a culinary arts and bakery programs based on her goals and interests. However, most of the culinary arts and bakery program required higher tuition fees and supplemental fees. Because her family could not afford the extra fees, she had to give up her own goals and enroll in the nursing program. Another participant (P#8, Female, Focus Group) also shared similar stories, saying,

I was planning to apply for the film major. But the department required [me] to have several expensive cameras and lenses, a high-quality computer, as well as the extra tuition fees for fine art lectures. I don’t want to spend all the saving[s] of my family…I have to select a university major…which I hate…that can make money….

(P#8, Female, Focus Group)

In short, the East Asian perspective, social context, and social expectation about the nursing and medical professions always limited their opportunities and career choices [6,25]. Unlike many studies conducted in the westernized societies and communities, many Taiwanese nursing students decided to enroll into one of the nursing programs based the social and cultural expectations [21]. Although university major and career development should be a selection of individuals, such preferences are not widely available in eastern societies. Furthermore, securing financial resources for their parents and elderly always forced them to enter the medical profession due to the stable salary and other financial considerations [29]. Although registered nurses may earn a reasonable salary for their family, it is unfair for both registered nurses and patients in this case in Taiwan. Although career mismatching always happens due to various reasons, this study discovered some significant findings which may gradually be detrimental to the medical and social health care system [11].

### 3.2. Influences from the Participants’ Contextual Environment

#### 3.2.1. Parental Recommendations

“I do this degree for my parents” (P#13, Female, Interview). It is worth noting that the perspective of “I have to do this degree for my parents” was shared 320 times based on the transcripts.

Young adults and recent secondary school graduates usually do not have significant work and life experience from which to select a major that may, in turn, be an investment in their long-term career plans. One potential way to obtain recommendations is from their parents. Due to the ideas of collectivism, many East Asian people tended to conduct activities and behaviors based on the benefits of the groups and communities. Without the permissions from family members and group members, East Asian people usually conduct nothing further because of the ideas of respectfulness.

All advocated the ideas of collectivism with the traditional East Asian perspective [4] due to their respectfulness towards family members and elders in their cohort [25]. In fact, they consulted with their parents and even grandparents about university selection, academic major, and career development before they submitted their university application. One participant (P#20, Female, Interview) recalled a story about marking her major selections, saying,

My mother asked me to study nursing as both of my parents are medical professionals in the national-level hospital. Perhaps I am not a good medical profession as I do not have any passion for my patients. But I have to listen to my parents as they are the one who took care of my life. I have to be respectful to them as a good child…This is traditional of us…Although I hate nursing…you don’t know how much I hate nursing and the career development of a registered nurse…But I have to do this degree for my parents….

(P#20, Female, Interview)

Another participant (P#28, Female, Interview) also shared a similar situation at the same focus group activity section, saying,

My father is a doctor, and my mother is a nurse…they expected my sister and I [to] become medical practitioners…so, my sister was asked to enrol in a nursing school and so am I…my interest and career goal is to become a news reporter. But what can I do? Can I not listen to my parents? They spent almost half of their life for two of us…I cannot just say no to them…I hate nursing…But I am doing this degree for them…but for my own interests and goals….

(P#28, Female, Interview)

From the above sharing, many participants selected their major and career development based on their parents’ desires and recommendations instead of their own [29]. A study indicated that East Asian people tended to listen to their parents and cohort members as recommendations for their decision. Unlike people in the westernized society, East Asian people advocate the notion of respectfulness towards their parents, elders, and community members. Therefore, this study also reflected the practices of the current participants.

In addition to parental recommendations being an extension of the parents’ original occupations, a large number of participants advocated that they were asked to study a major that their parents had not been able to achieve during their own youth. In fact, these expectations from parents are not uncommon in the East Asian perspective [27]. During the last century, most of the East Asian countries and regions were developing as third-world countries. Therefore, youth and students at that time did not have chances for university education. Therefore, parents of this generation and even the society have a higher-level of expectations in the current social context. One participant (P14, Female, Focus Group) was one of many with this case, saying,

My mother was raised in a low-income family and wished to become a nurse during her childhood…but she couldn’t…now, she asked me to complete her dream. But she did not ask me if I want to do so. As a daughter, I hope I can complete her dream for the purpose of respectfulness…however, I can tell you that I hate nursing…I studied this nursing degree for my mother…I will not join the nursing profession afterwards…absolutely no….

(P14, Female, Focus Group)

Similarly, another participant (P#17, Female, Focus Group) also shared a case of a comparable situation, saying,

My parents did not go to university during their young age…[so they] forced me to go to university…during my early teenage [years], they asked me to [study] medical biology. I have no interest…but my mother liked this…I [studied]. Now, my parents want me to study nursing…I enrolled at my nursing programme now…because I want to be a good girl…but I am sure that I hate this nursing subject and I studies this degree for them obviously…I will not join this nursing profession afterwards…But I have to study this for my parents….

(P#17, Female, Focus Group)

After P#17 shared her experience in a focus-group activity section, another participant (P#18, Female, Focus Group) echoed her similar case, saying,

I am sure a lot of students selected their major based on some expectations of the East Asian perspectives…I am sure more than half of us [classmates]’ majors were selected by parents or someone at home…at least four women in my dorm room were…I am so surprised that four girls [roommates] in our room were forced to study nursing because of our parents’ decision…We won’t join the nursing profession afterwards as we all studied that for our parents…We all understood that we hate nursing so much….

(P#18, Female, Focus Group)

When the researcher asked questions about the interrelationships of financial responsibility, academic major, and career decision and development, one participant (P#42, Female, Interview) exclaimed that due to pressure and expectations from her parents,

I absolutely want to become an early childhood teacher…but my mother just wanted me to study nursing in the capital city…if I cannot complete the degree, who is going to take care of her late [in] life? I am just a machine to take care of the elderly. But I have to take care of her, due to the filial piety…this makes me hate nursing so much….

(P#42, Female, Interview)

One participant (P#25, Female, Interview) expressed that she had to listen to her parents’ suggestion to attend medical school instead of the school of agriculture, due to financial considerations. Based on her life stories, the participant showed a solid awareness of her interests and career goals in the farming industry. However, her sense of filial piety limited her arguments because her parents’ suggestions were a higher consideration than her own interests and career goals [31].

In short, all participants advocated that their major selections and career developments were chosen by their parents due to the East Asian perspective, social context, and social expectation, particularly parental recommendations and respectfulness [23,24,25,26,27,28,29,30,31,32,33,34]. The finding from this section was hardly found in much of the current literature with a westernized perspective. It is worth noting that all participants always respected their parents instead of their own interests and goals. Although respectfulness is encouraged, such mismatching may damage the health and social care system.

#### 3.2.2. Teachers’ Recommendations

In addition to the recommendations from parents, teachers also controlled the participants’ decisions about their university enrollment, academic major, and career development. Nearly all participants revealed that their secondary school principal and homeroom teacher[s] had forced them to go to a nursing program because their enrollment would increase the reputation of the secondary school. One participant (P#23, Female, Interview) shared,

My secondary school is located in a rural community…I am the first graduate who receive[d] a first-rated GSAT score…[my] school principal asked me to go to the capital city and stud[y] a bachelor’s degree in nursing science, so the secondary school can promote my name and achievement in the city hall. I have asked my parents, family member[s], teachers, counsellors, and social workers about this. All of them advocated the relocation to the capital city and nursing school…It seems like I have no choice…but I want to study ocean studies….

(P#23, Female, Interview)

Teachers’ recommendations did not influence just a single student’s decision, but a large number of participants. Questions about teachers’ recommendations were asked during both the individual and focus-group activity sections, and nearly two-thirds of the participants shared their opinions on how teachers’ recommendation influenced their decision. Several significant opinions are listed below. For example, a participant (P#39, Female, Interview) shared negative experience, saying,

…my 12th grade homeroom teacher told me that the shortage of medical doctor[s] would be terminated within a decade. So I listened and obeyed her opinion and switch[ed] to nursing. This is the worst recommendation so far in my life…I want to be a doctor…not a nurse…I hate nursing, I like to be a medical doctor as my career development…It looks like I am studying for my teacher…I am so angry and upset….

(P#39, Female, Interview)

Another participant (P#22, Female, Interview) also told her lived stories between her teachers and herself to the focus group participants, saying,

…my teacher asked us to fill up the application form in front of her. She assigned us with the particular subject[s]. If we [did] not listen to her, I think that would be a little bit irresponsible? So I just listened to her. But this is certainly not my own will…So like, I am studying this degree for my teacher or what?

(P#22, Female, Interview)

In short, nearly all participants exhibited filial piety and respectfulness to their teachers and other school professionals [23,24,25,26,27,28,29,30,31,32,33,34]. Although some of them argued that their university enrollment and academic major, and their career decisions, were not their first choice, they felt that as good students they had to show the required filial piety and respectfulness.

#### 3.2.3. Pressures from Family Members

Besides family and teachers who had daily interactions with the participants, other family members, such as cousins, uncles, and aunts, also revealed expectations about the participants’ decisions regarding their university enrollment, academic major, and career pathway. Almost all participants expressed experience in this subtheme. One participant (P#3, Female, Focus Group) shared that her decisions were forced by her uncle, saying,

…my uncle told my mother that he used to work in a hospital and believed the nurses can make a lot of money. So, he highly recommended [to] my mother…[that] I select this direction. My mother and my uncle sent me the nursing brainwashing messages everyday…respectfulness, listening, orders from the parents…I am sure I hate nursing now…but I am studying this degree exclusively for these two people….

(P#3, Female, Focus Group)

Another participant (P#1, Female, Focus Group) also added to this theme and said,

…my uncle is [in] upper leadership in chained clinics in the capital city. He always convinced my mother, and I worked for him. So, he called my mother every day and asked me to study nursing…nursing is a subject that I hated…But the pressure from my uncle and mother…I have to follow this pathway…for them….

(P#1, Female, Focus Group)

During the same focus group activity section, Participant (P#29, Focus Group) shared a negative experience, saying,

I was the only one who was forced by other family members…in the Taiwanese society, many people believe being a doctor or medical practitioner is an excellent occupation. I agreed. But this is not something that I want to do for my whole life. I always thought I was tricked by my family as well as the society….

(P#29, Focus Group)

In short, many participants expressed negative opinions and experiences about the influence of family [23,24,25,26,27,28,29,30,31,32,33,34]. Because East Asian people tend to respect their elders’ recommendations [29], nearly all participants followed the wills of their family members instead of their own personal interests. It is worth noting that not only parents but also related family members, had the authority to order the participant to choose a particular direction for study and career choice.

#### 3.2.4. Comparison with Other Relatives

Many participants seemed to have had common experiences about how their parents compared their (i.e., the participants’) university enrollment, major, and even GSAT scores with those of other children with similar backgrounds. When one of the participants (P#19, Female) was asked how she had decided to start her undergraduate study in nursing, she said that her parents compared her GSAT score and university application form with those of other children who were living in the same neighbourhood. The following remarks made by some of the participants seem to show similar situations of negative experiences. For example, a participant (P#16, Female, Interview) shared,

My mother asked [about] other people’s academic major selections in the community centre. Afterwards, she came back and asked me to study that nursing…I was very surprised that my career and major are decided and selected by a group of aunts in the community centre…I hate nursing for sure…But as a Taiwanese girl…I needed to listen to our cohort and members of my family…If I don’t, I cannot show the respectfulness to the community….

(P#16, Female, Interview)

Another participant (P#1, Male, Interview) also shared that his mother brought his application form to the community centre and asked opinions from other children. Besides sending their information and report cards to the community centre, some participants told the researcher their parents had even brought all their family members and relatives to their home for discussion. One participant (P#23, Female) shared her experience, saying,

During the application period, my parents called everyone to my home for suggestions and discussions. My elder cousins provided more than 20 suggestions. My parents always compare[d] my scores, university enrolment, and major intentions to everyone…they compared my scores and major…my score was not excellent. Some of them even laughed…after they laughed, I still had to follow my mother’s decision….

(P#1, Male, Interview)

Another participant (P#26, Female, Interview) shared a similar situation, saying,

My GSAT score was high enough to apply [for] most of the appropriate majors. I would like to go to a university in the southern part of Taiwan, as I wanted to escape from my family…but my parents wanted me to study near them. So I stayed…my parents wanted me to study medicine. Even if I want to study nursing, I have to follow their wills…because they are my parents….

(P#26, Female, Interview)

Last but not least, the cohort and community-based collectivism always influenced how East Asian people behave. In this case, although the participants have their own goals and interests for major and career development, they did not have many choices due to the perspective of collectivism and respectfulness from their elders, family members, and members in their community. The results of this study outlined the relationship between the East Asian perspective, social context, and social expectation. The researcher used nursing students as a sample to explore and discover how this relationship exists in contemporary society. Without a doubt, many behaviors, ideas and perspectives were influenced by this relationship [23,24,25,26,27,28,29,30,31,32,33,34].

Attention should be paid to the directives given by the parents and family members. Although filial piety is a traditional aspect of the East Asian culture that may not change in the short term in Taiwan, the opinions and expressions of youth should be respected [36]. It is important to note that individuals’ academic and career interests and goals significantly influence their career pathways and career development [58,59]. Parents and family members should avoid ordering their children to base their decisions about university enrollment and university subject selections on the desires of their elders. One participant (P#41, Female, Interview) indicated that playing a musical instrument as an academic major was her desire because that hobby was developed based on the decisions of her parents. However, she had no choices due to her parents’ decision and application. Therefore, parents should also learn that they need to release more authority to their children and that the children should be taught career development navigation, such as vocational skills training [60].

## 4. Conclusions

To the best of the knowledge, this is one of the very first nursing studies that is based on the approach of the relationship between an East Asian perspective, social context, and social expectation for East Asian university students’ decision-making about their major selections and career developments. In some other countries, in which individuals do not have the significant sense of filial piety and obedience toward their parents and elders that East Asians do, individuals may follow their own interests and career goals for university enrollment and selection of their academic major, particularly in the field of nursing education [1,7,16].

In addition to the East Asian educational systems and structures that exist at the macro-level, at the micro-level filial piety and family negotiation continue to play a vital role for young adults in their decision-making about university enrollment, academic majors, and career progression, and that role could influence their long-term career investment and development [23,24,25,26,27,28,29,30,31,32,33,34]. Based on the data and experiences shared by this study’s 42 participants, all students indicated that their decisions and intentions were solid but were made unwillingly. However, due to their sense of filial piety, almost all of them had tended to listen to and obey suggestions or orders from their parents, teachers, and elders [35].

Some readers may argue that parents accept opinions from the youth in contemporary society. However, because this study was conducted recently in Taiwan with several dozen young adults, it seems clear that their sense of filial piety in the decision-making process is not likely to change in the short run. In general, secondary school graduates are usually under the age of 18 years old. Therefore, their university applications and related forms must be signed by parents or guardians. Although their sense of filial piety would not change even if the students had reached the age of 18 years old, young university students in their early adulthood should have greater authority to decide their own career pathways. The study’s findings indicate that some young adults choose to avoid the dictates of filial piety and instead pursue their own interests and career goals. For those interviewed here, the pursuit of their own interests and career goals combined with those directed by filial piety sometimes occurred [20,35,36,37]. For example, they may have pursued a double university subject major and minor study, to also satisfy their own desires.

### 4.1. Limitations

Every research study has its limitations. Two limitations have been found in this study. First, this study was a qualitative research study with 42 traditional-age nursing students. However, as mentioned before, nursing education and career development are famous career selections for many university students and second-career changers. Therefore, future research may expand the population to non-traditional age students, second-career changers, returning students, and adult students.

### 4.2. Implementations

In recent decades, some vocational higher education institutions have created vocation-oriented undergraduate degree programs for students who want to develop hands-on skills for their career pathways [61]. In addition to vocational undergraduate degree programs, some educational systems also allow individuals to take their first and second year of university education with a university subject as undecided. Both of those educational elements are significant blueprints for reforming Taiwanese and even all East Asian higher education institutions and universities, with the goal of expanding the current curriculum and policies for students with non-traditional backgrounds.

Instead of changing the sense of filial piety, the universities, departments of education, and related agencies could reform specific policies in order to avoid putting limitations on young adults. As discussed above, some participants expressed that the university policy did not allow them to switch their university subject. However, recent graduates of secondary school and young adults usually do not have enough life experience to choose appropriate career pathways during their late teenage years. Thousands of university graduates do not participate in the industry or subject area in which they received their undergraduate degree. As a result, if universities would allow enrolled students to switch their academic major after their registration and enrollment, that potentially could increase the graduation rate and interest levels of students and contribute to their learning and enjoyment. It is also worth noting that the relevant agencies should discuss creating an undecided academic major for students who encounter difficulty deciding on a specific major.

Parents also can learn skills and techniques for sharing short-term, middle-term, and long-term career goals and academic interests with their children, while providing the children with the appropriate rewards, appreciation, encouragement, support, and involvement. In addition, parents can learn the significance of helping their children explore and discover their own academic interests and career goals, by bringing them on field trips to university campuses and organizations, introducing them to various cultural backgrounds and diversities, and increasing their understanding of particular subject matters. By conducting these parenting steps, the parents can help their children to exercise filial piety by a show of respectfulness toward their parents, while at the same time making their own decisions about university enrollment, university subject selection, and career development.

## Figures and Tables

**Table 1:** A list of themes and subthemes for this study.

Themes and Subthemes		
**3.1.**	**Influences from the Participants’ Contextual Environment**	
	3.1.1.	The East Asian Perspective on Occupational and Role Expectations
	3.1.2.	Academic Results
	3.1.3.	Financial Influence
**3.2.**	**Influences from the Participants’ Family and Elders**	
	3.2.1.	Parental Recommendations
	3.2.2.	Teachers’ Recommendations
	3.2.3.	Pressures from Family Members
	3.2.4.	Comparison with Other Relatives

## Appendix A

Interview Protocol Questions: The First Interview

(1) Why do you want to study nursing as your bachelor’s degree major? Please tell me more.
(2) Do you enjoy your major? Why or why not?
(3) How did you select your university major during secondary school?
(4) If you can select it again, would you select nursing as your major?
(5) How would you think filial piety for your university major selection and other related behaviors?
(6) Further questions will be asked as follow-up questions.

## Appendix B

Interview Protocol Questions: The Second Interview

(1) Let’s think about the biggest reasons why would you select nursing as your major?
(2) Without this reason, would you select nursing again?
(3) Who select this major (i.e., nursing)? Yourself, families, friends, peers influence, parents etc.?
(4) Would other people influence your major selection/career development/university choice etc.?
(5) Would you think filial piety take some positions in the selection process?
(6) Further questions will be asked as follow-up questions.
